# Data-Driven Phenotyping Reveals Nonuniform Association Between Age and Mortality After Aortic Surgery: Retrospective Cohort Study of UK Biobank Data

**DOI:** 10.2196/75611

**Published:** 2026-03-11

**Authors:** Maria Elisabeth Leinweber, Fadi Taher, Miriam Kliewer, Afshin Assadian, Amun Georg Hofmann

**Affiliations:** 1 Department of Vascular and Endovascular Surgery, Klinik Ottakring, Montleartstraße 37, Vienna, 1160, Austria, 43 1491504103

**Keywords:** aneurysm, aortic aneurysm, cluster analysis, survival analysis, outcomes

## Abstract

**Background:**

Life expectancy and age are frequently considered factors to assess perioperative and postoperative mortality risks in patients affected by aortic pathologies, which can affect the decision whether to suggest invasive treatment.

**Objective:**

This study aims to investigate the association between age and all-cause mortality after invasive aortic treatment.

**Methods:**

Unsupervised clustering (k-means) using data from the UK Biobank was conducted for patients with aortic pathologies (*International Classification of Diseases, Tenth Revision* [ICD-10] group I71) receiving endovascular or open surgical treatment. Clustering variables encompassed demographic and clinical parameters. Survival analyses (postoperative survival time in days to all-cause death) between clusters and cluster-derived age groups were conducted.

**Results:**

The study included 1801 individuals undergoing surgical or endovascular repair for aortic aneurysms. Unsupervised cluster analysis identified distinct groups primarily based on age, both in models using 2 or 3 clusters. Clusters with older patients at surgery exhibited lower postoperative survival, with perioperative mortality disproportionately affecting these groups. While age was significantly associated with postoperative mortality overall (hazard ratio 1.07, 95% CI 1.05‐1.08), this association diminished in older clusters after excluding perioperative deaths, a trend confirmed in analyses adjusted for relevant confounders.

**Conclusions:**

Unsupervised cluster analysis revealed age as the primary factor distinguishing patient groups undergoing invasive treatment for aortic pathologies. However, age at surgery appears to have different consequences in certain age brackets, indicating a complex nonuniform relationship.

## Introduction

Aortic aneurysms (AAs) encompass a group of pathologies affecting the aorta in any of its segments from the ascending aorta to the infrarenal abdominal aorta, which is the most prevalent type [1]. Apart from true degenerative aneurysms, dissections are commonly included in this group of conditions or summarized as aortic syndromes due to a similar enlargement of diameter and treatment options [2]. The aorta is now considered an organ rather than a simple conduit to other tissues due to its complex structure and function [3]. Considering the high rate of adverse outcomes during acute ruptures, preemptive treatment is favored when possible in elective settings and is associated with significantly lower rates of perioperative morbidity and mortality [4]. Over the past 2 decades, shifts in global cardiovascular risk factors, the establishment of national screening programs, and the widespread adoption of endovascular aneurysm repair have significantly altered the epidemiology and management of AA [5-7]. Most high-income countries have experienced a marked decline in both incidence and mortality rates for elective and ruptured AA. However, demographic changes and expanded treatment options for older and frail patients have introduced new challenges [5,8].

Estimated life expectancy is frequently considered to be a relevant factor in clinical decision-making as the risks and benefits of treatment options may be assessed differently for patients with varying life expectancies. It is broadly suggested that younger and healthier patients can reasonably be exposed to the higher perioperative risks of open surgical repair to balance out limitations of endovascular strategies including concerns with durability and imaging surveillance [4,9]. The tradeoff between short-term survival advantages of endovascular repair and potential long-term disadvantages is not yet fully elucidated and, in general, is more nuanced and influenced by patient characteristics, expectations, anatomy, and comorbidities [10].

Analyses of large population-based datasets and administrative registries have identified several risk factors associated with adverse perioperative outcomes and impaired long-term survival. However, predicting mortality and serious adverse events is complex, and traditional association studies alone may not be sufficient [11-14].

Unsupervised clustering algorithms can be used to identify patterns within complex clinical data, enabling a different approach to risk stratification by grouping patients with similar phenotypes. Unlike traditional regression models, which isolate independent effects and may produce high hazard ratios for rare but extreme risks, cluster analysis can identify clinically coherent patient archetypes where multiple low and moderate risk factors can accumulate to define high-risk subgroups. This is particularly valuable in surgical cohorts, where multimorbidity, rather than selective patient factors, frequently determines outcomes.

In this work, we investigated whether an unsupervised cluster analysis based on demographic and clinical patient characteristics and naïve to postoperative outcomes can be leveraged to identify distinct postoperative survival patterns for specific patient groups.

## Methods

### Study Design and Dataset

In this retrospective observational analysis, an unsupervised cluster analysis was conducted using data from the UK Biobank [15]. The UK Biobank is a large population cohort study with 502,488 participants aged 40 to 69 years, with a recruitment phase from 2006 to 2010, spanning over 22 assessment centers in the United Kingdom. During recruitment, participants consented to synchronizing their UK Biobank patient file with their electronic medical records from the National Health Service (NHS) [16]. The World Health Organization (WHO)’s *International Classification of Diseases, Tenth Revision* (ICD-10) *codes* were used to code clinical diagnoses of hospital inpatient data. The *Office of Population, Censuses and Surveys: Classification of Interventions and Procedures* (OPCS-4) codes were used to code operations and procedures. Data from the NHS Information Center for participants from England and Wales and from the NHS Central Register Scotland for participants from Scotland were used to obtain date and cause of death [17,18]. The investigation was conducted in all patients with the ICD-10 group I71 (AA and dissection). Included OPSC-4 codes to classify procedures were: L18*, emergency replacement of aneurysmal segment of aorta; L19*, other replacement of aneurysmal segment of aorta; L25.4, operations on aneurysm of aorta, not elsewhere classified (NEC); L26.6, transluminal aortic stent graft with fenestration, NEC; L26.7, transluminal aortic branched stent graft, NEC; L27*, transluminal insertion of stent graft for aneurysmal segment of aorta; and L28*, transluminal operations on aneurysmal segment of aorta.

### Ethical Considerations

This study accessed UK Biobank data. Ethical approval for the UK Biobank was obtained from the North West Multi-Centre Research Ethics Committee (11/NW/03820). All enrolled participants gave written informed consent prior to enrollment in accordance with the tenets of the Declaration of Helsinki.

### Cluster Analysis

Cluster analysis was performed using the k-means clustering algorithm. Optimization of k was performed using the NbClust function (*NbClust* package [19]) in R (version 4.4.1; R Foundation for Statistical Computing), which uses 30 different indices. Clusters were formed based on 14 features, encompassing demographics and comorbidities. The list of variables included: sex, age at surgery, coronary heart disease, hypertension, heart failure, cardiac dysrhythmia, chronic kidney disease, BMI, smoking, peripheral aneurysms, history of cancer, aortic pathology type, aortic rupture, and type of surgery. Comorbidities and treatment were binary coded, sex was one-hot encoded, and aortic pathology was ordinal structured (dissections, thoracoabdominal, thoracic, and abdominal). Variables were chosen based on their effects on outcomes in aortic surgery [4] and life expectancy in general. Optimization suggested different k-values using the *NbClust* package with the same level of recommendation (5 metrics suggested 2 or 3 clusters each). Clusters were generated with the centers set to 2 and nstart set to 25. Optimization of k and cluster formation was carried out with a set seed. Since clustering requires no missing data, 18 (1%) out of the 1819 patients with aortic pathologies undergoing invasive treatment had to be excluded from the dataset. This was considered reasonable with a low risk of introducing significant selection bias.

### Sensitivity Analyses

Multiple steps were included in the analytical workflow to ensure the robustness of our findings. First, 2 different cluster analyses were conducted fitting 2 and 3 clusters to investigate whether the same set of variables would separate clusters from each other. Second, age cutoffs used in the cluster analyses were shifted to 65/75 years to investigate the consistency of the cluster-derived results. Third, survival analyses were done using different subsets of the study population, stratifying by clusters and after removing deaths within the perioperative period (30 d). Fourth, varying sets of covariates were introduced to adjust survival models and establish a set of parameters for the final model. Potential confounders included demographics such as age at surgery and sex, comorbidities used during clustering, smoking history, aortic pathology, as well as type and year of surgery.

### Statistical Analysis

Descriptive statistics were used to summarize baseline characteristics, with continuous variables reported as mean (SD) or median (IQR), depending on their distribution, and categorical variables presented as frequencies and percentages. Survival analyses (postoperative survival in days to all-cause death) were conducted using the Kaplan-Meier curves to estimate survival, stratified by clusters or indices derived from the clustering analysis. Log-rank tests were used to compare survival curves between groups. Cox regression models were used to fit survival models with varying covariates as mentioned above and Wald Test *P* value and 95% CI were assessed for each covariate. Statistical significance was defined as *P* value <.05. Cubic splines (4 degrees of freedom) were fitted based on the survival models to calculate and visualize age-related hazard ratios. All analyses were conducted using R. Multimedia Appendix 1 illustrates the analytic workflow and included packages [20-25]. The code outline can be accessed at the public GitHub repository [26] or in Multimedia Appendix 1. The actual analyses were conducted in the UK Biobank Research Analysis Platform.

## Results

### Study Population

The study included 1801 individuals, predominantly male (n=1500, 82.3%) with a median age at surgery of 69 (IQR 65‐74) years. Coronary heart disease and arterial hypertension were highly prevalent, affecting 56.6% (n=1019) and 81.5% (n=1468) of participants, respectively. Heart failure and cardiac dysrhythmia were observed in 36.7% (n=661) and 53.1% (n=956) of cases. Peripheral aneurysms occurred in 12.3% (n=222), and 82% (n=1477) reported a history of smoking. AAs were primarily abdominal (n=1204, 66.9%), followed by thoracic (n=394, 21.9%) and thoracoabdominal (n=9, 0.5%), with dissections reported in 10.8% (n=194). Ruptures were noted in 9.9% (n=179) of cases (Table 1). In summary, 29.5% of patients did not survive the observational period.

**Table 1.** Demographic and clinical characteristics of the study population (N=1801).

Characteristics	Total (N=1801)
Sex, n (%)	
Female	301 (16.7)
Male	1500 (82.3)
Age at surgery, mean (IQR)	69 (65-74)
Coronary heart disease, n (%)	1019 (56.6)
Arterial hypertension, n (%)	1468 (81.5)
Heart failure, n (%)	661 (36.7)
Cardiac dysrhythmia, n (%)	956 (53.1)
Chronic kidney disease, n (%)	726 (40.3)
BMI, median (IQR)	28.0 (25.6‐30.9)
Peripheral aneurysms, n (%)	222 (12.3)
Smoking (ever), n (%)	1477 (82)
History of cancer, n (%)	229 (12.7)
Aortic aneurysm, n (%)	
Abdominal	1204 (66.9)
Thoracic	394 (21.9)
Thoracoabdominal	9 (0.5)
Dissection	194 (10.8)
Rupture	179 (9.9)

### Unsupervised Cluster Analysis

Cluster analysis was performed fitting 2 and 3 cluster solutions (see “Methods” section). When fitting 2 clusters, age appeared to be the primary factor separating groups. Cluster 1/2 had a younger median age at surgery (62, IQR 58-65 y) compared to cluster 2/2 (73, IQR 70-76 y). Both clusters had similar rates of arterial hypertension (approximately 81%) and smoking (approximately 82%). Differences included a higher prevalence of cardiac dysrhythmia and chronic kidney disease in cluster 2/2 (55% and 42.8%, respectively) compared to cluster 1/2 (49.3% and 35.5%, respectively), corresponding to the higher age. Abdominal aneurysms predominated in both clusters, but cluster 1/2 showed a higher frequency of dissections (14.7% vs 8.7%) and strictly thoracic aneurysms (26.8% vs 19.3%), while ruptures were comparable (approximately 10%; Table 2)

**Table 2.** Fitting 2 clusters to the study population. Cluster 1/2 consists of younger patients (at the time of surgery) with more thoracic pathologies.

Variable	Cluster 1/2 (n=612)	Cluster 2/2 (n=1189)
Female (%), proportion (95% CI)	14.9 (12.0‐18.4)	17.7 (15.6‐19.9)
Age at surgery, median (IQR)	62 (58-65)	73 (70-76)
Coronary heart disease (%), proportion (95% CI)	58.8 (54.8‐62.7)	55.4 (52.5‐58.2)
Arterial hypertension (%), proportion (95% CI)	81.2 (77.8‐84.2)	81.7 (79.4‐83.8)
Heart failure (%), proportion (95% CI)	37.7 (33.9‐41.7)	36.2 (33.5‐39.0)
Cardiac dysrhythmia (%), proportion (95% CI)	49.3 (45.3‐53.4)	55 (52.1‐57.8)
Chronic kidney disease (%), proportion (95% CI)	35.5 (31.7‐39.4)	42.8 (40.0‐45.6)
BMI, median (IQR)	28.1 (25.5‐31.3)	2 (25.6‐30.6)
Peripheral aneurysms (%), proportion (95% CI)	12.7 (10.1‐15.9)	12.1 (10.4‐14.1)
Smoking (ever) (%), proportion (95% CI)	80.4 (76.9‐83.5)	83.7 (81.5‐85.7)
Connective tissue disease (%), proportion (95% CI)	3.1 (1.9‐5.0)	3.4 (2.5‐4.6)
Aortic aneurysm		
Abdominal, %	57.8	71.5
Thoracic, %	26.8	19.3
Thoracoabdominal, %	0.7	0.4
Dissection, %	14.7	8.7
Rupture (%), proportion (95% CI)	9.2 (7.0‐11.9)	10.3 (7.0‐11.9)

When grouped into 3 clusters, age differences were more pronounced: cluster 1/3 (n=700; median age at surgery 75, IQR 73-78 y), cluster 2/3 (n=811; 68, IQR 65-69 y), and cluster 3/3 (n=290; 57, IQR 53-60 y). Cluster 3/3 had the highest rates of heart failure (43.8%) and thoracic aneurysms (34.1%) and the lowest smoking prevalence (73.5%). Cluster 1/3 had the highest aortic rupture rate (12%), with abdominal aneurysms being most common (73.9%). Cluster 2/3 displayed intermediate characteristics across many variables, including rupture rate (8.8%) and the distribution of aortic pathologies (Table 3). Due to the predominant separation between clusters by age, this was further evaluated. It was identified that the first cluster analysis split patients at age 66/67 years, while the second cluster analysis used 61/62 years and 71/72 years as thresholds. All patients below or above the respective thresholds were exclusively grouped in the corresponding clusters.

**Table 3.** Fitting 3 clusters in the study population. Cluster 1/3 includes the oldest patients at the time of surgery. Cluster 3/3 contains the youngest patients who have a higher rate of cardiac comorbidities and more thoracic pathologies.

Variable	Cluster 1/3 (n=700)	Cluster 2/3 (n=811)	Cluster 3/3 (n=290)
Female (%), proportion (95% CI)	18 (15.3‐21.0)	15.4 (13.0‐18.2)	17.2 (13.3‐22.0)
Age at surgery, median (IQR)	75 (73-78)	68 (65-69)	57 (53-60)
Coronary heart disease (%), proportion (95% CI)	52.1 (48.4‐55.9)	59.6 (56.1‐62.9)	59 (53.2‐64.5)
Arterial hypertension (%), proportion (95% CI)	82.7 (79.8‐85.3)	81 (78.2‐83.6)	80 (75.0‐84.3)
Heart failure (%), proportion (95% CI)	36.1 (32.6‐39.8)	34.6 (31.4‐38.0)	43.8 (38.1‐49.6)
Cardiac dysrhythmia (%), proportion (95% CI)	56.4 (52.7‐60.1)	49.2 (45.7‐52.7)	55.9 (50.1‐61.5)
Chronic kidney disease (%), proportion (95% CI)	45.7 (42.0‐49.5)	37.9 (34.6‐41.3)	34.1 (28.9‐39.8)
BMI, median (IQR)	28.0 (25.7‐30.5)	28.0 (25.4‐31.1)	28.3 (25.3‐31.3)
Peripheral aneurysms (%), proportion (95% CI)	13.6 (11.1‐16.5)	10.9 (8.9‐13.3)	13.4 (9.8‐18.1)
Smoking (ever) (%), proportion (95% CI)	83.4 (80.5‐86.0)	85.1 (82.4‐87.4)	73.5 (68.1‐78.3)
Connective tissue disorder (%), proportion (95% CI)	2.9 (1.8‐4.5)	7.4 (5.8‐9.4)	5.2 (3.1‐8.5)
Aortic aneurysm			
Abdominal, %	73.9	67.7	46.9
Thoracic, %	17.7	21.1	34.1
Thoracoabdominal, %	0.4	0.5	0.7
Dissection, %	7.7	10.7	18.3
Rupture (%), proportion (95% CI)	12.0 (9.7‐14.7)	8.8 (6.9‐11.1)	8.3 (5.6‐12.1)

### Cluster Survival Analyses

**Figure 1.**
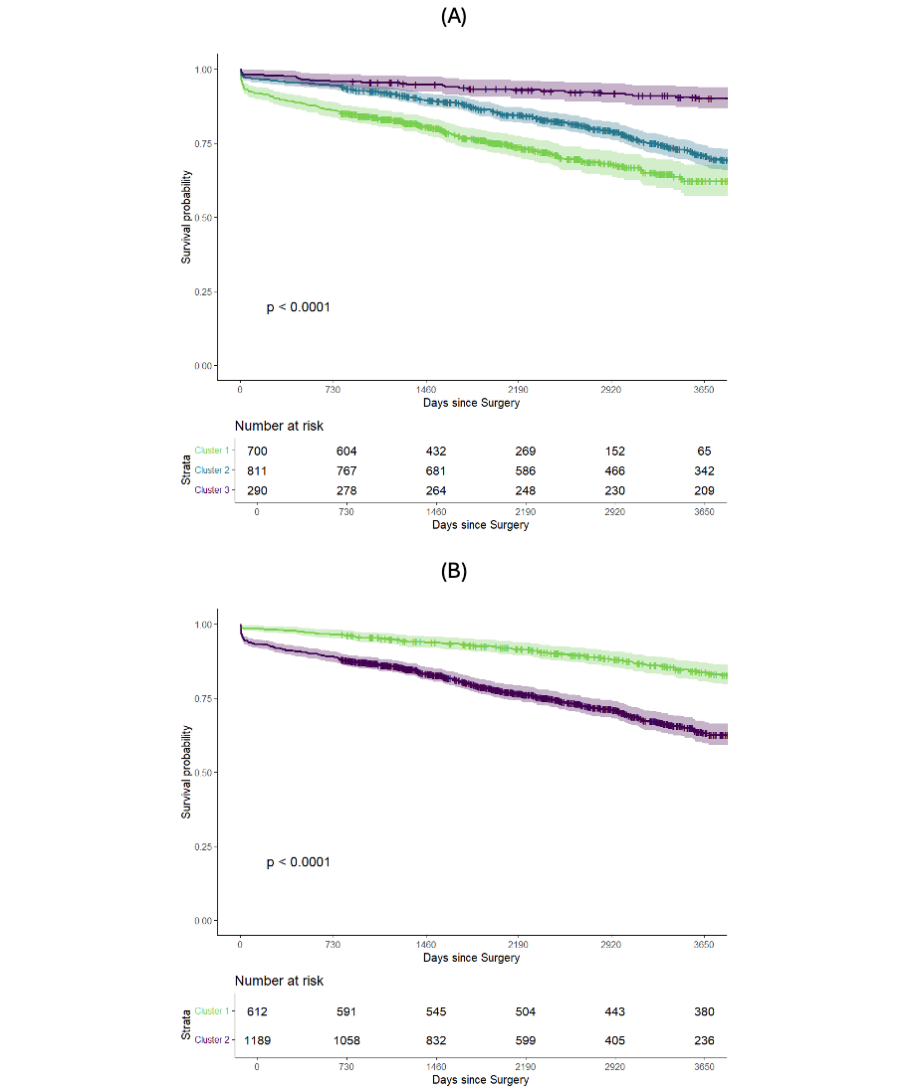
Kaplan-Meier curves illustrating postoperative survival for each cluster. Clusters with older patients at surgery had lower postoperative survival rates. (A) 3 clusters and (B) 2 clusters. The depicted *P* value refers to a log-rank test.

Survival analyses were conducted for each cluster analysis. In both cases (2 or 3 clusters), age at death was higher in patients undergoing treatment at a later age, reflective of the selection bias of age at treatment. However, postoperative survival was significantly lower in these clusters (clusters 1/3 and 2/2; Figures 1A and 1B). It appeared that the main phase of separation in both cases lay within the perioperative period, where older clusters were associated with a higher all-cause mortality. Especially when comparing clusters 2/3 and 3/3, differences in long-term mortality appeared to stabilize between clusters.

**Figure 2.**
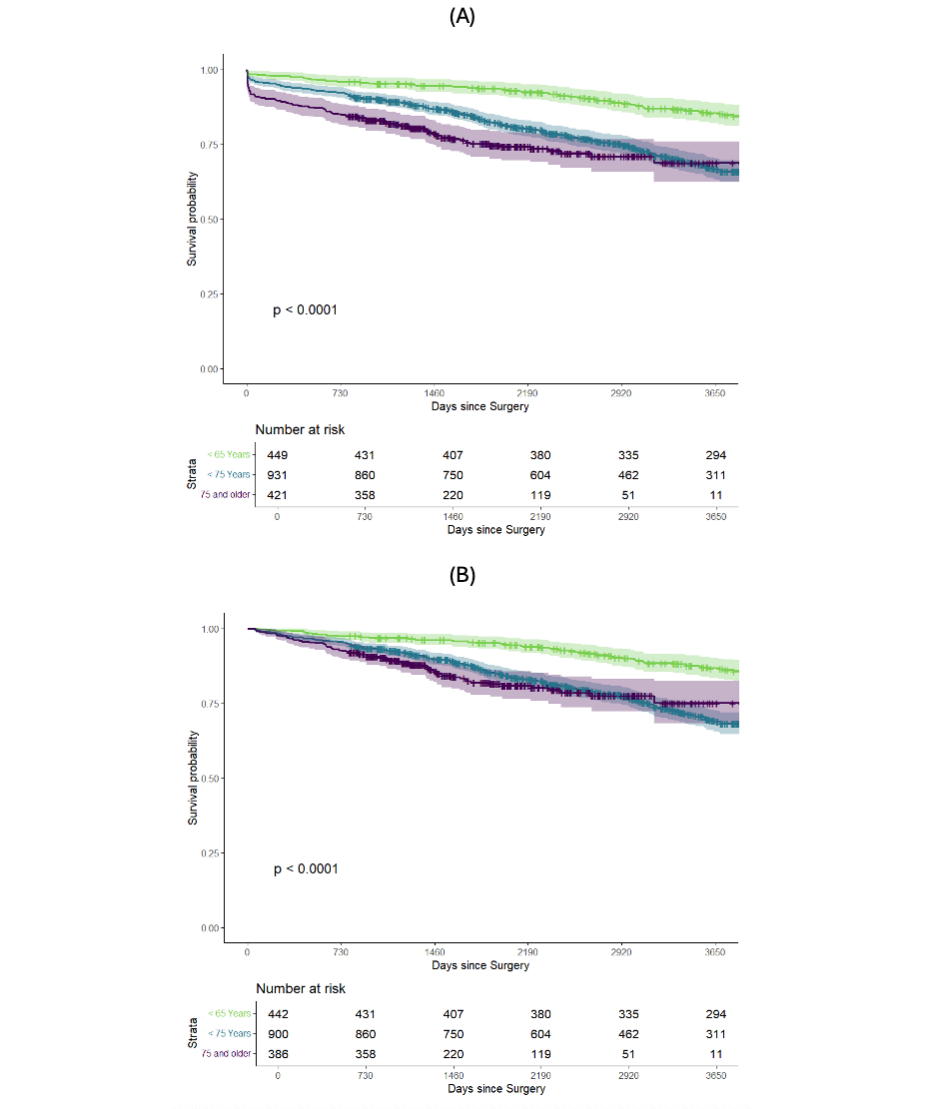
Kaplan-Meier curve illustrating postoperative survival stratified by age. Patients with higher age at surgery have lower postoperative survival rates. (A) Full observational period and (B) after removing the first 30 postoperative days. The depicted *P* values refer to a log-rank test.

To explore whether these observations would remain constant after shifting age brackets, the study population was divided into 3 subgroups at 65 and 75 years. Postoperative survival was repeatedly lower in older age groups that were characterized by a higher perioperative mortality (Figure 2A). However, after removing deaths within the first 30 postoperative days, the survival estimates of all groups not only converged but, especially regarding the 2 older age groups, there was no evidence for a difference in all-cause mortality rates (Figure 2B).

### Age and Mortality

To further explore the relationship between age and postoperative survival, univariate survival analyses were conducted in different subsets of the study population (Table 4). In the full study population, age was significantly associated with increased all-cause mortality after invasive aneurysm treatment. When stratifying by clusters, this association faded for the cluster with the oldest patients (cluster 1/3), indicating that in very old patients, additional years of age do not significantly increase mortality risks. However, since the perioperative period was associated with disproportionate mortality in older people, the analyses were repeated removing patients who died during the first 30 postoperative days. Now, there was no evidence for an association between all-cause mortality and age in both cluster analyses for the oldest clusters (cluster 2/2 and cluster 1/3).

**Table 4.** The association between age at surgery and mortality in varying patient groups in a single-predictor survival analysis (N=1801).

Group	Participants (n)	Coefficient	95% CI	*P* value^a^
Study population	1801	1.07	1.05‐1.08	<.001
Cluster 1/2	612	1.06	1.03‐1.09	<.001
Cluster 2/2	1189	1.04	1.01‐1.07	.008
Cluster 1/3	700	1.02	0.96‐1.07	.55
Cluster 2/3	811	1.07	1.02‐1.12	.008
Cluster 3/3	290	1.08	1.03‐1.13	.002
Study population (without first 30 d)	1728	1.06	1.05‐1.08	<.001
Cluster 1/2 (without first 30 d)	604	1.07	1.03‐1.10	<.001
Cluster 2/2 (without first 30 d)	1124	1.02	0.99‐1.06	.19
Cluster 1/3 (without first 30 d)	653	0.97	0.90‐1.03	.32
Cluster 2/3 (without first 30 d)	790	1.06	1.01‐1.12	.02
Cluster 3/3 (without first 30 d)	285	1.08	1.03‐1.13	.003

a The *P* value is derived from the Wald test.

Subsequently, the analyses were repeated adjusting for the year of surgery since overall mortality rates have decreased over time and this was therefore considered a relevant confounder (Table 5). There was again strong evidence for an association between age and post-operative mortality, except for cluster 1/3 after deaths were removed in the perioperative period. To fit a final model, varying sets of confounders were introduced, and nonsignificant parameters were removed. The final analyses adjusted for year of surgery, chronic kidney disease, heart failure, and type of aortic pathology (Table 6). However, there was strong evidence for an association between postoperative mortality and age in most subgroups. However, after excluding perioperative deaths, age was no longer statistically significantly associated with all-cause mortality in either cluster 1/3 or cluster 3/3 (the youngest cohort).

**Table 5.** The association between age at surgery and mortality in varying patient groups in a survival analysis adjusted for year of surgery (N=1801).

Group	Participants (n)	Coefficient	95% CI	*P* value^a^
Study population	1801	1.08	1.06‐1.11	<.001
Cluster 1/2	612	1.06	1.03‐1.09	<.001
Cluster 2/2	1189	1.10	1.06‐1.15	<.001
Cluster 1/3	700	1.08	1.00‐1.15	.04
Cluster 2/3	811	1.11	1.05‐1.17	<.001
Cluster 3/3	290	1.07	1.02‐1.12	.004
Study population (without first 30 d)	1728	1.08	1.07‐1.11	<.001
Cluster 1/2 (without first 30 d)	604	1.07	1.03‐1.10	<.001
Cluster 2/2 (without first 30 d)	1124	1.10	1.06‐1.15	<.001
Cluster 1/3 (without first 30 d)	653	1.06	0.97‐1.15	.18
Cluster 2/3 (without first 30 d)	790	1.10	1.04‐1.17	<.001
Cluster 3/3 (without first 30 d)	285	1.07	1.02‐1.13	.004

a The *P* value is derived from the Wald test.

**Table 6.** The association between age at surgery and mortality in varying patient groups in a survival analysis adjusted for year of surgery, chronic kidney disease, heart failure, and type of aortic pathology (N=1801).

Group	Participants (n)	Coefficient	95% CI	*P* value^a^
Study population	1801	1.09	1.05‐1.14	<.001
Cluster 1/2	612	1.04	1.01‐1.08	.02
Cluster 2/2	1189	1.09	1.05‐1.14	<.001
Cluster 1/3	700	1.08	1.00‐1.15	.04
Cluster 2/3	811	1.10	1.04‐1.16	<.001
Cluster 3/3	290	1.05	1.00‐1.11	.04
Study population (without first 30 d)	1728	1.07	1.05‐1.10	<.001
Cluster 1/2 (without first 30 d)	604	1.05	1.01‐1.08	<.001
Cluster 2/2 (without first 30 d)	1124	1.09	1.05‐1.14	<.001
Cluster 1/3 (without first 30 d)	653	1.07	0.98‐1.16	.13
Cluster 2/3 (without first 30 d)	790	1.09	1.03‐1.15	.002
Cluster 3/3 (without first 30 d)	285	1.05	1.00‐1.11	.06

a The *P* value is derived from the Wald test.

**Figure 3.**
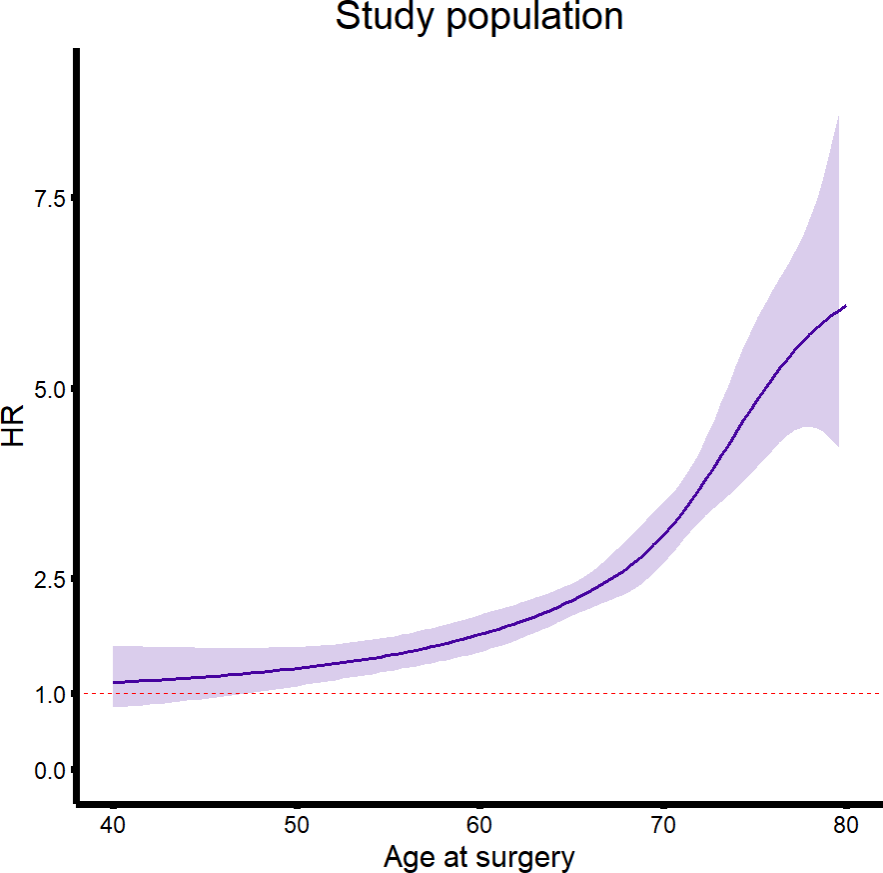
Age-specific hazard ratios (HRs) for (A) all-cause mortality after invasive aortic treatment for the whole study population, (B) after removing deaths in the perioperative period (first 30 d), and (C) concerning mortality in the first 30 days after surgery. The shaded area indicates the 95% CI.

Figure 3A-C illustrate the age-specific hazard ratios for all-cause mortality and the total study population, after removing perioperative deaths, and exclusively for death during the perioperative period.

## Discussion

The study analyzed 1801 individuals undergoing open surgical or endovascular repair for AAs. The majority were male (82.3%) with a median age of 69 years. Cardiovascular comorbidities and smoking were highly prevalent. AAs were primarily abdominal (66.9%). Overall, 29.5% of patients did not survive the observational period. Unsupervised cluster analysis revealed distinct groupings exclusively based on age. In a 2-cluster model, the clusters were separated at a median age of 62 and 73 years. A 3-cluster model further stratified the population, with median ages of 57, 68, and 75 years. Survival analyses highlighted lower postoperative survival in older clusters relative to treatment, with perioperative mortality disproportionately impacting these clusters. There was strong evidence that age was significantly associated with all-cause mortality overall but showed diminished significance in older clusters after excluding perioperative deaths. Adjusting for confounders confirmed this observation, with age losing significance in the youngest and oldest clusters in later analyses.

That clusters were primarily fitted based on age is not necessarily intuitive from a physician’s perspective. We would have expected the presence of ruptures and the specific type of pathology to exert a larger influence on cluster formation, as these factors all have a relevant influence on clinical practice. Interestingly, the strictly data scientific approach of the analysis attributed heterogeneity between the included patients predominantly to age at the time of surgery. We hypothesize that a combination of collinearity, for example, a higher rate of dissections on younger patients, and homogeneity, with similarly distributed comorbidities, could explain this finding. Furthermore, aortic surgery, which is by definition a high-risk procedure, may homogenize the investigated patient sample (at least for the electively treated patients) as patients must prove a certain level of health to qualify for such an operation. Age is strongly associated with postoperative mortality following invasive treatment of aortic pathologies, but there is solid evidence that this relationship varies across age groups. In the older clusters, the diminished association between age and all-cause mortality after excluding perioperative deaths suggests that perioperative risk plays a critical role in the age-related mortality disparity. Specifically, older patients demonstrated disproportionately higher all-cause mortality within the first 30 days after surgery, implying that the perioperative period is a key driver of mortality in this subgroup. This complies with previous evidence that reported a perioperative survival disadvantage after invasive aneurysm treatment in older patients [27,28].

This differential impact across age groups highlights the complexity of the relationship between age and mortality risk, which does not appear to be linear. While overall mortality increases with age, the cluster analysis revealed thresholds or transition zones in risk rather than a simple uniform progression. The higher perioperative mortality in older patients might reflect their greater vulnerability to surgical stress, emphasizing the importance of targeted perioperative management in this group. Additionally, the lack of a strong age-mortality association in the older clusters after the perioperative period suggests that factors beyond age, such as the type of aneurysm or co-existing conditions, may have a greater influence on long-term survival in these populations. Overall, the data support a nuanced interpretation of age as a risk factor, driven largely by perioperative mortality. Investigating nonlinear relationships between age and outcomes such as mortality in biomedical research has been of growing interest in the previous years [29-32]. This work contributes to the cumulative body of evidence that might gradually induce a paradigm shift in patient risk stratification.

From a statistical perspective, the findings reflect a nonlinear association in which the hazard function for age is dominated by early postoperative events in older patients, leading to attenuation of the age-mortality relationship once perioperative deaths are removed. This pattern is consistent with threshold-like behavior rather than a single global linear term, suggesting that conventional models assuming proportional and constant age effects may obscure important variation across the age distribution.

The clinical implications of this work emphasize the need for tailored perioperative management strategies that account for age-related differences in mortality risk. The necessity to identify solutions for an aging population is not confined to the field of healthcare, but is a subject of considerable interest within it. It is estimated that by 2050, 129.8 million individuals in Europe will be aged 65 years or older [33], with significant implications for the demand on national health care systems and the surgical workforce [34-36]. Therefore, the question of age as a limitation for operative procedures such as AA repair has attracted increasing attention in vascular surgery [27] but is discussed in other fields such as orthopedics with knee arthroplasty [37] as well. A substantial body of research has indicated an elevated risk of perioperative complications for older patients across various fields, including common abdominal [38-40] and vascular procedures [41,42]. In addition to adverse outcomes, surgical treatment in older patients has been demonstrated to be more resource-intensive, characterized by prolonged hospital stays [35,43]. The unsupervised cluster analysis identified distinct age thresholds at surgery, providing a framework for stratifying patients into risk groups. This approach moves beyond arbitrarily defined age-based subgroups, such as octogenarians or nonagenarians, which have traditionally been used as proxies for elderly populations in surgical research and have been previously questioned regarding their use [44].

However, the sensitivity analysis showed that alternative age cutoffs at 65 and 75 years produced similar Kaplan-Meier survival curves, suggesting that the transitions between age-based clusters are fluid rather than strictly defined. Consequently, these thresholds should not be interpreted as rigid cutoffs but rather as approximate transition zones that may guide clinical decision-making. For example, patients in the older age groups are at increased risk for perioperative mortality, highlighting the need for enhanced perioperative care, such as optimization of cardiovascular and respiratory function, and close monitoring during the immediate postoperative period. Similarly, younger patients may benefit from strategies aimed at mitigating long-term risk factors, such as smoking cessation and management of hypertension.

The identification of fluid but meaningful thresholds by the cluster analysis provides a valuable tool for clinicians, balancing the need for structured risk stratification with the recognition of patient-specific variability. From a group-level perspective, comorbidities were rather homogenously distributed among clusters. This may be due to case selection, as the analysis included only patients deemed suitable for surgery. As a result, eligibility for surgical treatment could have a homogenizing effect on this patient sample. Nevertheless, on an individual scale, this is certainly not the case and also requires attention when evaluating invasive treatment for a given patient. Age at surgery can give some guidance when evaluating perioperative and postoperative risks, and they vary for distinct age groups, but other factors still impose meaningful effects on survival as well.

The reliance on observational data introduces the potential for selection bias, particularly evident in the older clusters, where longer median survival age after surgery may simply reflect the fact that these patients underwent surgery at a later age. This delayed treatment may be indicative of slower disease progression, possibly due to reduced exposure to risk factors such as smoking or poorly controlled hypertension, but it complicates direct comparisons between age groups. Additionally, the UK Biobank lacks detailed information on aneurysm morphology, including diameters and growth rates, which are critical for understanding disease severity and progression. Such missing data may limit the granularity of risk stratification and affect the interpretation of clinical outcomes. Data from the UK Biobank are also subject to a volunteer selection bias. Furthermore, the use of administrative and diagnostic coding introduces the possibility of errors or misclassification in identifying aneurysm type, surgical interventions, and comorbid conditions. Future studies incorporating more comprehensive clinical data and prospective designs could address these limitations and further refine the understanding of age-related risks in aortic aneurysm surgery. This will also address the need for further external validation of these findings.

This study highlights age as one of the key factors in postoperative all-cause mortality following AA surgery, with older patients experiencing higher perioperative mortality. Unsupervised cluster analysis revealed age thresholds that guide risk stratification reflecting transitions in age-related mortality risk.

## Supplementary material

10.2196/75611 Multimedia Appendix 1 R code analytic workflow.

## Abbreviations

AA: aortic aneurysm
ICD-10: *International Classification of Diseases, Tenth Revision*
NEC: not elsewhere classified
NHS: National Health Service
OPCS-4: *Office of Population, Censuses and Surveys: Classification of Interventions and Procedures*
WHO: World Health Organization
